# The Incidence and Relative Risk of Stroke among Patients with Bipolar Disorder: A Seven-Year Follow-Up Study

**DOI:** 10.1371/journal.pone.0073037

**Published:** 2013-08-30

**Authors:** Hung-Chi Wu, Frank Huang-Chih Chou, Kuan-Yi Tsai, Chao-Yueh Su, Shih-Pei Shen, Tieh-Chi Chung

**Affiliations:** 1 Department of Addiction Science, Kai-Syuan Psychiatric Hospital, Kaohsiung City, Taiwan; 2 Department of Community Psychiatry, Kai-Syuan Psychiatric Hospital, Kaohsiung City, Taiwan; 3 Department of Nursing, Meiho University, Ping-Tong County, Taiwan; 4 Graduate Institute of Health Care, Meiho University, Ping-Tong County, Taiwan; 5 Department of Nursing, I-Shou University, Kaohsiung City, Taiwan; National University of Singapore, Singapore

## Abstract

**Objective:**

This study aimed to estimate the incidence and relative risk of stroke and post-stroke all-cause mortality among patients with bipolar disorder.

**Methods:**

This study identified a study population from the National Health Insurance Research Database (NHIRD) between 1999 and 2003 that included 16,821 patients with bipolar disorder and 67,284 age- and sex-matched control participants without bipolar disorder. The participants who had experienced a stroke between 1999 and 2003 were excluded and were randomly selected from the NHIRD. The incidence of stroke (ICD-9-CM code 430–438) and patient survival after stroke were calculated for both groups using data from the NIHRD between 2004 and 2010. A Cox proportional-hazards model was used to compare the seven-year stroke-free survival rate and all-cause mortality rate across the two cohorts after adjusting for confounding risk factors.

**Results:**

A total of 472 (2.81%) patients with bipolar disorder and 1,443 (2.14%) controls had strokes over seven years. Patients with bipolar disorder were 1.24 times more likely to have a stroke (95% CI = 1.12–1.38; p<0.0001) after adjusting for demographic characteristics and comorbid medical conditions. In addition, 513 (26.8%) patients who had a stroke died during the follow-up period. The all-cause mortality hazard ratio for patients with bipolar disorder was 1.28 (95% CI = 1.06–1.55; p = 0.012) after adjusting for patient, physician and hospital variables.

**Conclusions:**

The likelihood of developing a stroke was greater among patients with bipolar disorder than controls, and the all-cause mortality rate was higher among patients with bipolar disorder than controls during a seven-year follow-up period.

## Introduction

Bipolar disorder is a major psychiatric disorder that is associated with several medical conditions contributing to substantial morbidity and mortality [1], [2]. Several studies have demonstrated common medical illnesses comorbid with bipolar disorder, including obesity, hyperlipidemia, hypertension and diabetes mellitus, all of which are recognized as risk factors for stroke [3].

When the authors reviewed the studies discussing the relationship between mood disorder and stroke, several mentioned the relationship between major depressive disorder and stroke. One study found that the presence of depressive symptoms is a strong risk factor for stroke in men but not in women [4]. Another study showed that depressive symptoms were an independent risk factor for the incidence of stroke/transient ischemic attack (TIA) in individuals <65 years of age [5]. Other studies have shown that depressive symptoms predicted stroke and that a significant relationship exists between depressive symptoms and stroke mortality [6], [7]. However, there were few studies that noted a relationship between bipolar disorder and stroke.

To the best of our knowledge, there have only been two cohort studies that have discussed factors related to stroke in patients with bipolar disorder. One study used a Danish registry dataset to estimate the risk among patients previously discharged with an affective disorder before receiving a stroke diagnosis. The results did not demonstrate an association between manic/bipolar disorder and stroke [8]. Another study used the Taiwan National Health Insurance Research Database (NHIRD) and estimated the risk of developing stroke among patients with bipolar disorder for the 6 years after hospitalization for an acute mood episode [9]. The results demonstrated that patients with bipolar disorder were twice as likely to develop stroke as patients undergoing an appendectomy during that same period. However, without using outpatient data, these results might not account for patients with less severe bipolar disorder (or stroke, or both), and using patients undergoing an appendectomy as a control group may limit the generalizability of these findings.

The present study therefore aimed to estimate the risk of stroke and the subsequent all-cause mortality rate in patients with bipolar disorder using the NHIRD admission and outpatient data across a seven-year follow-up period. The patients were compared to a randomly selected group of age- and sex-matched control individuals. The related factors of developing stroke for these two cohorts were subsequently calculated and compared after adjusting for demographic characteristics and comorbid medical conditions.

## Methods

### Participants

The National Health Insurance Program was established in Taiwan in 1995. It has enrolled up to 99% of the Taiwanese population and contracted with 97% of the medical providers [10]. The data for this study are from the 1999–2010 NHIRD in Taiwan. To verify the accuracy of the diagnosis, the National Health Insurance Bureau of Taiwan randomly reviews the charts of one out of 100 ambulatory and one out of 20 inpatient claimed cases and interviews patients. Since the NHIRD consists of anonymous public data released for research, this study conformed to the ethical standards established by the 2004 Declaration of Helsinki and was approved by the Institutional Review Board (IRB) at Kaohsiung Municipal Kai-Syuan Psychiatric Hospital (KSPH-2012–25) in Taiwan.

According to the International Classification of Disease, 9th Revision, Clinical Modification (ICD-9-CM) diagnostic criteria, the diagnostic coding of the Taiwanese Bureau of National Health Insurance (BNHI) was performed. The completeness and accuracy of the NHIRD has been affirmed by the Taiwan Department of Health and the Bureau of NHI through audit [11]. Patients with any type of severe mental disease, including mood disorders (ICD-9-CM code 296), may apply for BNHI catastrophic illness registration (CIR) cards in Taiwan to receive long-term treatment benefits. To qualify for a CIR card, a patient must be diagnosed by a specialist such as a psychiatrist. Patients pay only 10% of their medical expenses when they are hospitalized in Taiwan, and patients with a CIR card do not have to pay any medical expenses. Consequently, having a CIR card makes it relatively easy to obtain medical services [12] and the specialist evaluates the applicant according to the diagnostic criteria as carefully as possible before the BNHI will issue a CIR card.

**Figure 1:**
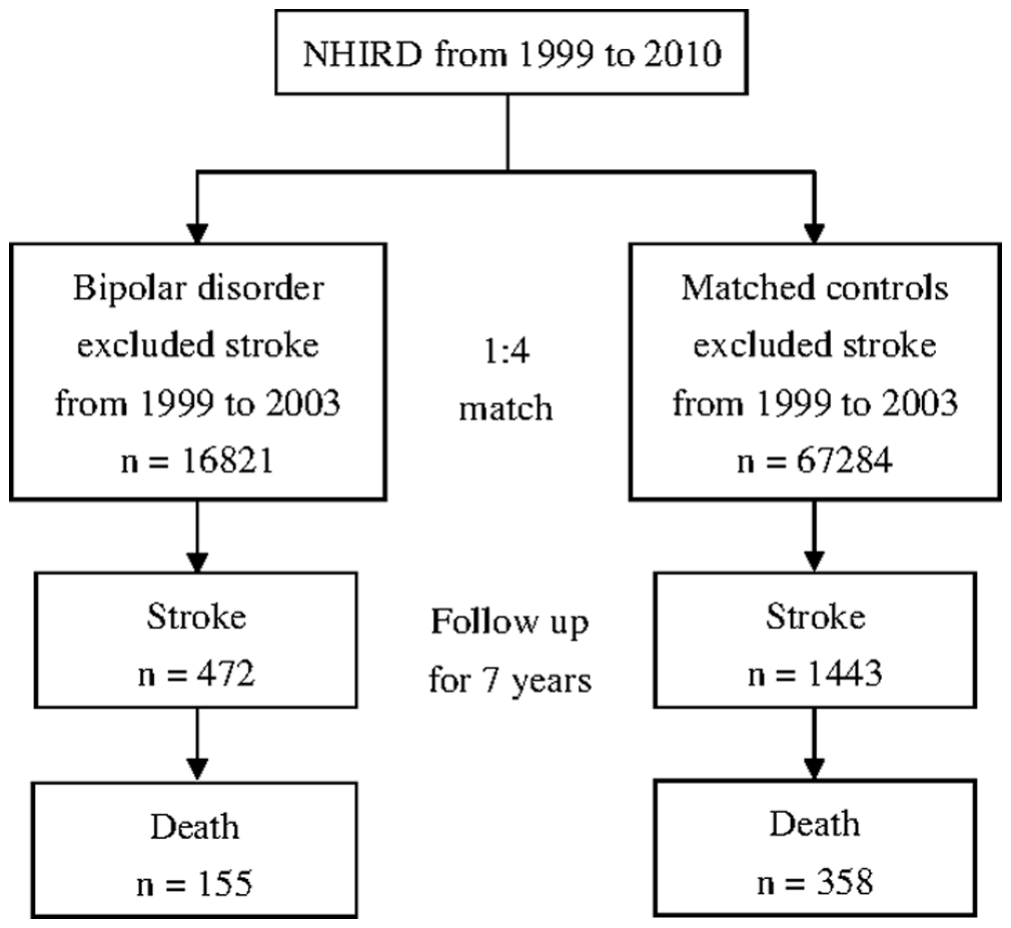
Sampling from the NHIRD.

The stability of the diagnosis of bipolar disorder was assured by the CIR. First, all patients with bipolar disorder who possessed CIR cards were included and all patients with a previous diagnosis of stroke (ICD-9-CM codes 430–438) were excluded. The study identified 16,821 patients with bipolar disorder (ICD-9-CM code 296.0X, 296.4X, 296.5X, 296.6X, 296.7X, 296.80 or 296.89), but not major depressive disorder (ICD-9-CM code 296.2X, 296.3X), in their CIR files between 1999 and 2003. From the NHIRD database that includes 23,981,020 participants, the authors excluded participants who had experienced a stroke between 1999 and 2003 or had suffered from bipolar disorder between 1999 and 2010. Then, 67,284 (1∶4) individuals were randomly selected as a control group and were matched to the patient group for age and sex with SAS software (SAS Institute Inc., SAS/STAT user's guide, Version 6, 4th edition, Cary, NC: SAS Institute Inc., 1989), as shown in Fig. 1.

### Measurements

The independent variables included age, sex, enrollee category (EC), level of urbanization and stroke comorbidities, which were directly obtained from the NHRID. EC is an important risk factor for stroke as a proxy measure of socioeconomic status [13]. The patients with bipolar disorder were classified into four subgroups: EC 1 (civil servants, full-time, or regular paid personnel with a government affiliation), EC 2 (employees of privately owned institutions), EC 3 (self-employed individuals, other employees, and members of the farmer’s or fishermen’s associations), and EC 4 (veterans, members of low-income families, and substitute service draftees) [14]. A previous study reported that stroke is associated with the level of urbanization [15]. The level of urbanization was determined by the population density, percentage of residents with a college or higher education, percentage of residents >65 years of age, percentage of residents who were agriculture workers, and number of physicians per 100,000 individuals. The level of urbanization was divided into urban, suburban, and rural settings. Previous studies have found positive associations between stroke and hypertension, diabetes, coronary heart disease (CHD), and hyperlipidemia [16]. Therefore, these comorbidities and the extracted details on medical comorbidities from the 1999 to 2003 administrative claimed data were accounted for in the following models.

The patients who suffered from stroke between 2004 and 2010 were diagnosed by neurologists, and the administrative codes were collected from the NHIRD. According to the ICD-9-CM codes, the types of stroke include hemorrhagic (ICD-9-CM codes 430–432), ischemic (ICD-9-CM codes 433–435), and unspecified (ICD-9-CM codes 436–438) stroke. The comorbidities of each patient were based on the Charlson Comorbidity Index Score (CCIS), which is widely used for risk adjustment in insurance claims datasets. The authors modified the CCIS to calculate the sum of weighted scores based on the relative mortality risk for 19 conditions [12], [17]. The CCIS was originally developed in 1987 by Mary Charlson and colleagues, who studied the 1-year all-cause mortality in a cohort of more than 500 patients admitted to the medical unit of a teaching hospital [18]. This index has been validated for predicting the mortality risk associated with a wide range of medical conditions, and it constitutes one of the most commonly used comorbidity indices to date. The physical illness information was extracted from data in the NHRID from 1999 to 2003.

We categorized hospitals by ownership status (e.g., public, private, or private non-profit), accreditation level (medical center [>500 beds], regional [250–499 beds] or district hospital [21–249 beds]), geographical location (North, Central, South, or East Taiwan), and whether they were teaching hospitals. A hospital’s accreditation level can be used as a proxy for both its size and its technological capabilities.

We followed the selected 16,821 patients with bipolar disorder and 67,284 controls from 2004 to 2010 to estimate the incidence of stroke and the survival time after the onset of stroke. The first dependent variable was the incidence of stroke between 2004 and 2010 in the bipolar disorder and control cohorts in this study. To evaluate the association between bipolar disorder and stroke, chi-squared tests and independent *t*-tests were used to examine the differences between these cohorts in terms of their socio-demographic characteristics and comorbidities. A patient was no longer a part of the study after having the first stroke. The Cox proportional-hazards regression was used to identify variables that predicted the length of time until a patient’s first stroke after controlling for age, gender, level of urbanization, EC and comorbidities using a forward stepwise selection with the likelihood ratio criterion (inclusion/exclusion criteria: *P*≤0.05/*P*>0.10, respectively). We tested whether the time dependent and continuous variables fit the proportional hazard assumption. If not, they were categorized to fit the assumption. The second dependent variable was the survival time of stroke patients between 2004 and 2010. We linked all study participants who were diagnosed with a stroke to the mortality data extracted from the “severe disease” file. Since the primary outcome was death, the risk evaluation commenced at the diagnosis of stroke, whereas the follow-up period ended either at death or on December 31, 2010. The other Cox proportional-hazard regression was used to compute the adjusted seven-year case fatality. Previous studies indicated that patients with bipolar disorder experience disparities in specialized medical care and have higher mortality rates [19]; therefore, the model accounted for this finding as well as other factors that are associated with the risk of patient mortality, such as patient age and sex, type of stroke, comorbidities, EC, level of urbanization, physician age and sex, caseload, and hospital characteristics and used a forward stepwise selection with the likelihood ratio criterion (inclusion/exclusion criteria: *P*≤0.05/*P*>0.10, respectively). We linked and analyzed all of the data using SAS software and set the significance level at α = 0.05.

## Results

A total of 16,821 bipolar disorder patients and 67,284 non-bipolar disorder controls were identified from the NHRID date for the period of 1999 to 2003. During the years from 2004–2010, 472 (2.81%) patients with bipolar disorder and 1,443 (2.14%) controls had strokes. Furthermore, 155 (32.84%) bipolar disorder patients and 358 (24.81%) controls died after the onset of stroke during the seven-year follow-up period.

Compared to the matched controls, patients with bipolar disorder were more likely to have diabetes, hypertension, hyperlipidemia, and CHD. Moreover, they were more likely to have a lower socioeconomic status and to live in rural areas than the matched controls (Table 1).

**Table 1:** Demographic characteristics and comorbid medical disorders of patients with bipolar disorder and matched controls.

	Patients with bipolar disorder		Matched controls		p
	n = 16821	%	n = 67284	%	
	(Mean)	(SD)	(Mean)	(SD)	
Age (Mean ± SD)	(43.19)	(14.42)	(43.19)	(14.42)	1.0000
Sex					1.0000
Male	7707	45.82	30828	45.82	
Female	9114	54.18	36456	54.18	
CCIS					<0.0001
0	15372	91.39	64288	95.55	
1	941	5.59	1668	2.48	
≥2	508	3.02	1328	1.97	
Diabetes					<0.0001
Yes	634	3.77	1095	1.63	
No	16187	96.23	66189	98.37	
Hypertension					<0.0001
Yes	669	3.98	1541	2.29	
No	16152	96.02	65743	97.71	
Hyperlipidemia					<0.0001
Yes	253	1.50	380	0.56	
No	16568	98.50	66904	99.44	
Coronary heart disease					<0.0001
Yes	375	2.23	739	1.10	
No	16446	97.77	66545	98.90	
Enrollee category					<0.0001
EC1	1543	9.17	6833	10.16	
EC2	4967	29.53	30548	45.40	
EC3	4927	29.29	19546	29.05	
EC4	5384	32.01	10357	15.39	
Level of urbanization					<0.0001
Urban	10741	63.85	42809	63.62	
Suburban	4580	27.23	19142	28.45	
Rural	1500	8.92	5333	7.93	

In all, 472 (2.81%) patients with bipolar disorder and 1,443 (2.14%) controls had strokes. After adjusting for confounding risk factors, the hazard ratio (HR) for having a stroke during the seven-year follow-up was 1.24 times greater for patients with bipolar disorder than for the matched controls (Table 2). There was also a greater likelihood of stroke among male patients, older patients, and patients of lower socioeconomic status. As expected, participants with diabetes, hypertension, and hyperlipidemia were more likely to have a stroke, and there is no significant collinear relationship among DM, hypertension and hyperlipidemia; however, CHD was not associated with stroke in this regression model.

**Table 2:** Adjusted hazard ratio of developing stroke during the seven-year follow-up period for patients with bipolar disorder and the matched controls (n = 84105).

	Adjusted HR	95% CI	p
Age	1.07	1.071-1.078	<0.0001
Sex (Male = 1, Female = 0)	1.34	1.224-1.466	<0.0001
Diabetes	2.07	1.769-2.412	<0.0001
Hypertension	1.56	1.352-1.807	<0.0001
Hyperlipidemia	1.52	1.169-1.965	0.002
EC (EC1 as reference)			
EC2	1.15	0.959-1.383	0.131
EC3	1.40	1.176-1.668	<0.0001
EC4	1.25	1.042-1.502	0.016
Level of urbanization (Urban as reference)			
Suburban	1.15	1.035-1.267	0.008
Rural	1.07	0.925-1.241	0.358
Bipolar *(Bipolar = 1, Non-bipolar = 0)	1.24	1.120-1.383	<0.0001

* Bipolar patients who suffered from stroke, n = 472(2.81%).

Non-bipolar patients who suffered from stroke, n = 1443(2.14%).

The stroke patients with bipolar disorder were more likely to be young, to have lower socioeconomic status and to have a higher CCIS than the matched controls (Table 3). In the comparison of only stroke patients, the patients with bipolar disorder were more likely to have comorbid diabetes than healthy controls. The patients with bipolar disorder were more likely to have strokes that were classified as unspecified stroke than the non-bipolar disorder controls. The physicians who treated stroke patients with bipolar disorder were older than the physicians who treated patients without bipolar disorder. Finally, the hospitals that cared for stroke patients with bipolar disorder were more likely to be eastern, public, state-run, district hospitals and were less likely to be teaching hospitals.

**Table 3:** Patient, physician, and hospital characteristics of stroke patients with bipolar disorder and matched controls.

	Stroke patients withbipolar disorder		Stroke patients withmatched controls		p
	n = 472	%	n = 1443	%	
	(Mean)	(SD)	(Mean)	(SD)	
**Patient Characteristics**					
Age (Mean ± SD)	(59.85)	(12.54)	(61.52)	(13.02)	0.0142
Sex					0.0116
Male	202	42.80	714	49.48	
Female	270	57.20	729	50.52	
CCIS					0.0297
0	357	75.64	1172	81.22	
1	68	14.41	155	10.74	
≥2	47	9.96	116	8.04	
Diabetes					0.0061
Yes	76	16.10	163	11.30	
No	396	83.90	1280	88.70	
Hypertension					0.5781
Yes	77	16.31	220	15.25	
No	395	83.69	1223	84.75	
Hyperlipidemia					0.1232
Yes	21	4.45	43	2.98	
No	451	95.55	1400	97.02	
Coronary heart disease					0.7221
Yes	36	7.63	103	7.14	
No	436	92.37	1340	92.86	
Enrollee category					0.0123
EC1	42	8.90	113	7.83	
EC2	103	21.82	373	25.85	
EC3	191	40.47	639	44.28	
EC4	136	28.81	318	22.04	
Level of urbanization					0.1560
Urban	276	58.47	771	53.43	
Suburban	139	29.45	483	33.47	
Rural	57	12.08	189	13.10	
Hemorrhagic stroke					0.1211
Yes	82	17.37	298	20.65	
No	390	82.63	1145	79.35	
Ischemic stroke					0.3994
Yes	311	65.89	981	67.98	
No	161	34.11	462	32.02	
Unspecified stroke					0.0023
Yes	79	16.74	164	11.37	
No	393	83.26	1279	88.63	
Deceased					0.0006
Yes	155	32.84	358	24.81	
No	317	67.16	1085	75.19	
**Physician Characteristics**					
Age (Mean ± SD)	(41.15)	(8.41)	(39.48)	(7.91)	<0.0001
Sex					0.1788
Male	430	91.10	1283	88.91	
Female	42	8.90	160	11.09	
Stroke patient caseload	(81.09)	(97.84)	(87.12)	(86.20)	0.2317
**Hospital Characteristics**					
Geographic location					0.0001
North	179	37.92	570	39.50	
Central	128	27.12	393	27.23	
South	128	27.12	436	30.21	
East	37	7.84	44	3.05	
Ownership status					0.0045
Public	149	31.57	370	25.64	
Private for-profit	138	29.24	387	26.82	
Private for-non-profit	185	39.19	686	47.54	
Hospital accreditation level					<0.0001
Medical center	131	27.75	469	32.50	
Regional hospital	204	43.22	703	48.72	
District hospital	137	29.03	271	18.78	
Teaching hospital					<0.0001
Yes	378	80.08	1273	88.22	
No	94	19.92	170	11.78	

A total of 513 (26.8%) stroke patients died during this 7-year study. The all-cause mortality HR for patients with bipolar disorder was 1.28 (95% CI = 1.06–1.55) after adjusting for patient, physician and hospital variables (Table 4). The mortality risk significantly increased when patients were male (HR = 1.43), were older (≥65 years old vs. <65 years old, HR = 1.59), lived in rural areas (HR = 1.29) or had higher CCISs (≥2 vs. <2, HR = 1.86). Hemorrhagic and unspecific stroke had a higher mortality risk than ischemic stroke. The risk decreased when those with a higher stroke caseload treated the patients.

**Table 4:** Adjusted hazard ratio of death within the seven-year follow-up period after the onset of stroke (n = 1915).

	Adjusted HR	95% CI	p
**Patient Characteristics**			
Age(≧65years old = 1	1.59	1.329-1.903	<0.0001
vs.<65years old = 0)			
Sex (Male = 1, Female = 0)	1.43	1.197-1.701	<0.0001
Diabetes	1.49	1.171-1.886	0.001
CCIS(≧2 = 1	1.86	1.441-2.412	<0.0001
vs.<2 = 0)			
Type of stroke (Ischemic as reference)			
Hemorrhagic	2.50	2.040-3.064	<0.0001
Unspecific	1.40	1.084-1.803	0.01
Level of urbanization (Urban as reference)			
Suburban	0.92	0.757-1.125	0.425
Rural	1.29	1.003-1.657	0.047
*Bipolar (Bipolar = 1, Non-bipolar = 0)	1.28	1.056-1.549	0.012
**Physician Characteristics**			
Stroke-patient caseload	0.718	0.597-0.864	<0.0001
(≧85 = 1 vs. <85 = 0)			

* Deceased bipolar patients with stroke, n = 155(32.84%).

Deceased non-bipolar patients with stroke, n = 358(24.81%).

## Discussion

To the best of our knowledge, our study is the first attempt to evaluate the related factors of stroke among both inpatients and outpatients with bipolar disorder compared to matched controls after adjusting for patient demographic data, comorbid medical disorders and socioeconomic status. A previous study by Lin et al. showed that patients with bipolar disorder comorbid with alcohol or substance dependence had a higher risk of stroke [9]. However, the prevalence of alcohol dependence was shown to be 1.2% among patients with bipolar disorder and 0.0% among controls, and the prevalence of substance dependence was 0.7% among patients with bipolar disorder and 0.0% among controls, respectively. Because the treatment of substance or alcohol dependence is not covered by national health insurance in Taiwan, the physicians are not prone to record the ICD-9-CM code of substance or alcohol dependence. Therefore, the prevalence of substance or alcohol dependence might be underestimated according to the data of the NHIRD [20]. Instead, we used CCIS, which has been validated for a wide range of medical conditions, and EC as a proxy measure of socioeconomic status to predict mortality risk.

Bipolar disorder is associated with multiple medical comorbidities. Our results indicate that patients with bipolar disorder are more likely to have diabetes, hypertension, hyperlipidemia and CHD, which is consistent with previous studies [1], [21]. It has been well documented that the incidence of stroke may be associated with these four illnesses and that the incidence of stroke is higher in men and older individuals [9]. During the seven-year follow-up period, the incidence of stroke in patients with bipolar disorder and controls was 2.81% and 2.14%, respectively. A previous study by Lin et al. showed that stroke occurred among 2.97% of patients with bipolar disorder and 1.50% of patients undergoing appendectomy between 1998 and 2003 [9]. The incidence of patients with bipolar disorder and stroke in the previous study by Lin et al. is higher than our finding, which may be due to the different study samples and methods between the studies [9]. Our study includes both inpatients and outpatients with bipolar disorder; however, the previous study by Lin et al. selected only inpatients. The severity of bipolar disorder might be related to the use of antipsychotic drugs, which has previously been linked to increased risk of insulin resistance, lipid abnormalities and hypertension [22], all of which can result in vascular events such as stroke.

Bipolar disorder is also associated with many psychiatric comorbidities. Patients with bipolar disorder often have comorbid anxiety, substance or alcohol use disorder, personality disorder and eating disorders [23], [24], [25]. The administrative data are used to estimate the prevalence rates of psychiatric comorbidities according to the ICD-9-CM codes from 300 to 319, which show that the prevalence of anxiety, dissociative and somatoform disorders (ICD-9-CM code = 300) is 4.65%, which is statistically significantly higher among patients with bipolar disorder than matched controls. The prevalence rates of the other psychiatric diseases according to the ICD-9-CM codes are also statistically significantly higher among patients with bipolar disorder than matched controls. However, the prevalence rates of the ICD-9-CM codes from 300 to 319 are all below 5%, which indicate that clinicians might emphasize the principal diagnosis and psychiatric comorbidities might not be well diagnosed. In addition, the variables including all patients with the ICD-9-CM codes from 300 to 319 do not fit the proportional hazard assumption and do not influence our findings. Therefore, the psychiatric comorbidities will not be shown in this study.

We found that increased age and higher CCISs were related to an increased risk of death in patients who suffered strokes. Young patients with bipolar disorder were at a high risk of stroke, especially females. However, the HR of death for men increased by 0.43 after adjustment. The association of sex and stroke mortality varies across studies. One 10-year follow-up study showed that death after stroke was associated with older age, male sex, greater stroke severity, and diabetes, regardless of the cause of death [26]. The other cohort study showed that advanced age, male gender, low BMI, history of diabetes, history of hypertension and history of transfusion were associated with an increased risk of total stroke mortality [27]. However, there was one study that did not show a difference in mortality for sex [28]. The other American study indicated that the mortality rate was higher for women who were younger than 35 years of age and older than 85 years of age, whereas the rate was lower for women between 35 and 85 years of age compared to that of men in the same age category [29].

We also found that patients with bipolar disorder were more likely to have unspecified strokes compared to the control group. Previous studies showed that death was less commonly caused by ischemic strokes compared to intracranial hemorrhage (ICH) and undetermined strokes [30]. The other review article concluded that ICH strokes were more likely than ischemic strokes to lead to death [31], which was similar with our findings.

Our results show that bipolar disorder patients with strokes had higher all-cause mortality than the controls during the seven-year follow-up period. However, no study has investigated the post-stroke all-cause mortality among patients with bipolar disorder. Some studies investigated the post-stroke mortality among patients with schizophrenia, and the results were controversial [10], [32], [33]. Some studies found that lithium, valproate and lamotrigine, all of which are mood stabilizers used to treat bipolar disorder, had broad neuroprotective properties and may therefore be promising therapeutic agents for the treatment of neurodegenerative diseases, including stroke [34], [35], [36]. However, we found that the incidence and mortality of stroke were higher among patients with bipolar disorder than among matched controls. That is, the actual mechanisms contributing to the association between bipolar disorder and the subsequent development of stroke remain unclear. Many causes, such as unhealthy lifestyle, comorbid medical conditions, and the use of psychotropic medications, could all contribute to the increased risk of stroke among patients with bipolar disorder, which will need further investigation.

When considering hospital characteristics, our results show that the hospitals where the patients with bipolar disorder were treated for stroke were more likely to be eastern, public, district hospitals that were unlikely to train physicians, and these factors were associated with poor outcomes [1], [2]. Previous studies have indicated that patients with bipolar disorder experience inequities in specialized medical care [19], [37], and stroke patients with psychosis have lower rates of cerebrovascular arteriography and carotid endarterectomy [37]. Consequently, care-givers should pay more attention to the physical condition of patients with bipolar disorder and give them regular physical and laboratory examinations since these patients can have more difficulty caring for themselves compared to other types of patients.

## Limitations

First, although we included inpatients and outpatients of bipolar disorder for study, the patients with bipolar disorder who did not possess a CIR card were excluded from this study, and the lack of information on patients without a CIR card may limit the generalizability of these findings. Second, the severity of stroke or bipolar disorder was not included in this database, and the potential for a selection bias might exist regarding the incidence of stroke among patients with bipolar disorder. Third, the diagnoses of bipolar disorder, comorbid conditions, and the determination of healthy controls depended on ICD codes. However, the National Health Insurance Bureau of Taiwan randomly reviewed the charts and interviewed patients to verify the accuracy of these diagnoses. In addition, the Registry for Catastrophic Illness Patient Database of the NHIRD confirmed the diagnosis of bipolar disorder. Fourth, our data lack information on dietary habits, smoking, alcohol consumption, and body mass index; therefore, some possible selection bias may exist. Fifth, our data lack information on the co-occurrence of other systemic diseases and the use of antipsychotic drugs, mood stabilizers, antidepressants, other medications and possible drug-drug interactions, which may be confounding factors that could affect our findings. In addition to physical comorbidities such as diabetes, hypertension, hyperlipidemia and CHD, bipolar disorder is highly associated with heavy drinking and smoking, which are risk factors for stroke even among non-bipolar disorder patients [38], [39]. Sixth, although the Cox proportional-hazard regression provided the answers of HRs of stroke and death in this study, the method is not developed to estimate the mediating or moderator effects of the variables. However, use of other statistical methods that estimates mediating and moderator effects, e.g., path analysis, is possibly unsuitable because the NHIRD is mainly composed of categorical variables which are likely to bias the outcome if defined as continuous.

## Conclusions

We found that patients with bipolar disorder were at a significantly higher risk for stroke and post-stroke death from any cause after adjusting for demographic, socioeconomic and comorbid medical variables. Clinicians should pay more attention to patients with bipolar disorder to prevent stroke and decrease mortality. The survey of vascular risk factors and regular neurological examination may be needed for patients with bipolar disorder, especially for older patients with physical comorbidities.
